# Plant oxylipins: adaptation to environmental stresses and impact on mycotoxin contamination

**DOI:** 10.3389/fpls.2025.1739321

**Published:** 2026-01-20

**Authors:** Giovanni Di Pasquale, Letizia Ottaviani, Marco Camardo Leggieri, Paola Giorni, Adriano Marocco, Paola Battilani, Alessandra Lanubile

**Affiliations:** 1 Department of Sustainable Crop Production, Università Cattolica del Sacro Cuore, Piacenza, Italy; 2 University School for Advanced Studies IUSS Pavia, Pavia, Italy

**Keywords:** abiotic stress, CYP74 enzymes, gene editing, jasmonates, lipoxygenase, mycotoxins

## Abstract

Due to increasingly frequent changes in climatic conditions and global warming, plants consistently deal with severe weather events including extreme temperature variations, floods and drought. These abiotic stressors resulting from climate change weaken host crop resistance, making them more exposed to fungal disease insurgences and mycotoxin contamination. Oxylipins are major players in the plant-environment interaction. Their synthesis begins with the oxygenation of polyunsaturated fatty acids by lipoxygenases (LOXs) to generate fatty acid hydroperoxides that in turn are converted into a huge assortment of bioactive compounds by specialized cytochrome P450 enzymes, known as CYP74. In the present review we focus on recent advances concerning oxylipin biosynthesis and the phylogenetic relationships among the main key enzymes of the oxylipin pathway considering five monocot and dicot plant species. Moreover, new information regarding the role of these signaling molecules on the plant physiology in response to abiotic stress and mycotoxin occurrence are provided along with the application of clustered regularly interspaced short palindromic repeats (CRISPR)/CRISPR associated (Cas) (CRISPR/Cas)-based tools. Here, we report the intervention of *LOX*, *allene oxide synthase*, *OPDA reductase*, *JASMONATE* (JA) *resistant* and *JA ZIM domain* genes along with the accumulation of JA and its conjugates, 12-OPDA, ketols and green leaf volatiles in response to abiotic stress. The modulation of *LOX* genes and the production of several fatty acids, oxylipins and sphingolipids is also required against mycotoxin contamination.

## 1 Introduction

Climate change is a complex event that consists of temperature and weather shifts, strongly impacting plant life (Janni et al., 2024). Abiotic stressors, like heat, cold and drought, have intensified in recent decades, causing detrimental effect on crop yields and making host crops more vulnerable to fungal disease onset (Sharma et al., 2023). Mycotoxins are secondary metabolites synthesized by a broad assortment of fungi. Their production is greatly impacted by climate change and warmer temperatures are increasing the distribution, abundance and co-occurrence of mycotoxin producing fungal species (Lanubile et al., 2021a; Casu et al., 2024).

**Figure 1:**
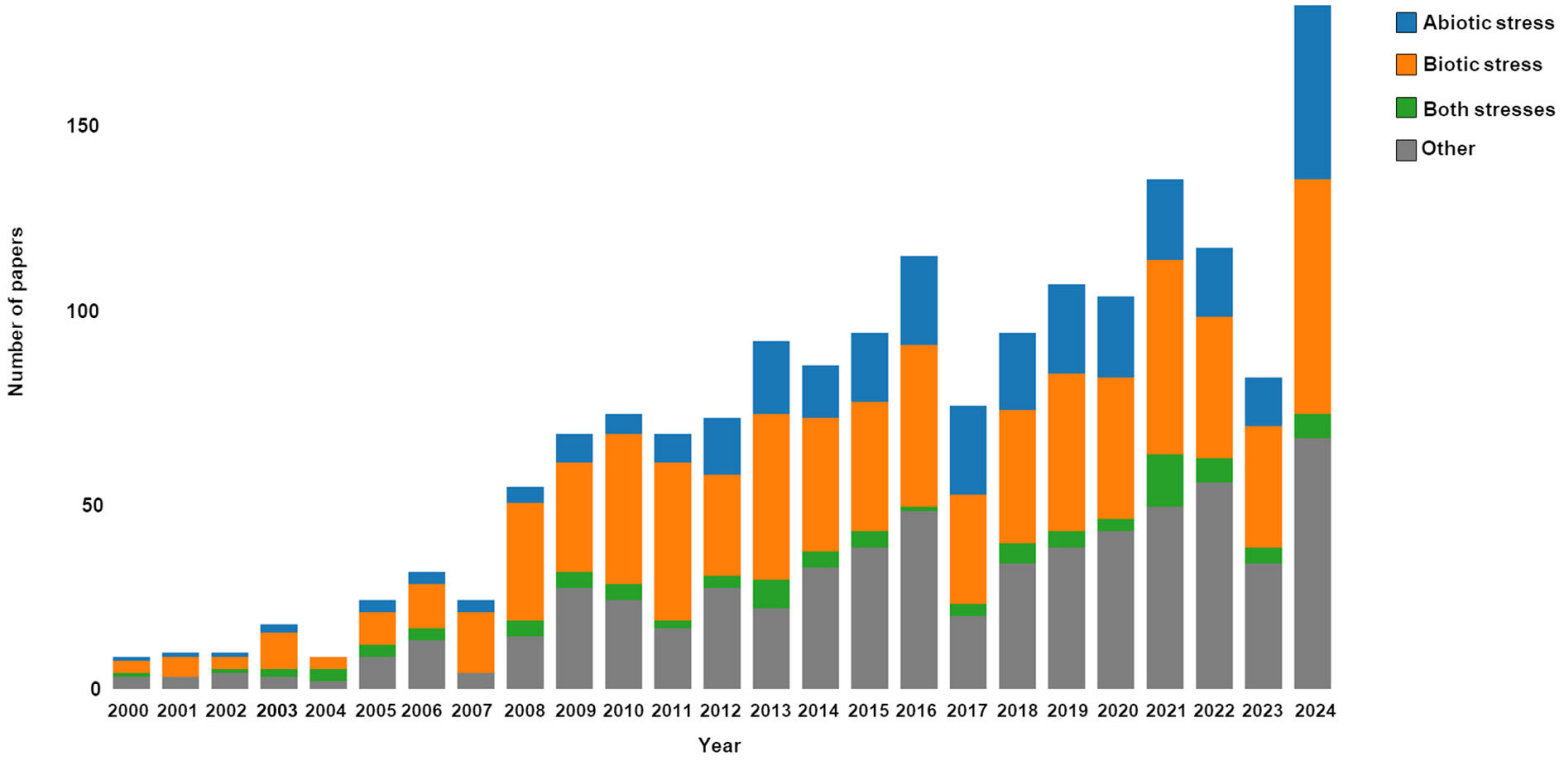
Number of articles published from 2000 to 2024 on the topic of plant oxylipins. The bar graph representing the terms related to plant oxylipins involved in abiotic stress is colored blue. The bar graph representing the terms related to plant oxylipins involved in biotic stress is colored orange. The bar graph representing the terms related to plant oxylipins involved in both abiotic and biotic stress is colored green. The bar graph representing the terms related to plant oxylipins involved in other physiological processes is colored grey. Terms were searched in Ovid and Scopus databases eliminating duplicated articles. The search was performed using the keywords “plant*” OR “crop*” AND “oxylipin*” OR “phyto-oxylipin*”. Records related primarily to medical, veterinary, mathematical, engineering, or non-biological sciences were excluded to maintain the focus on biological/agricultural areas. Additional filters were applied to remove papers centered on humans and other animals, duplicated papers, non-English papers, and to consider only research articles.

**Figure 2:**
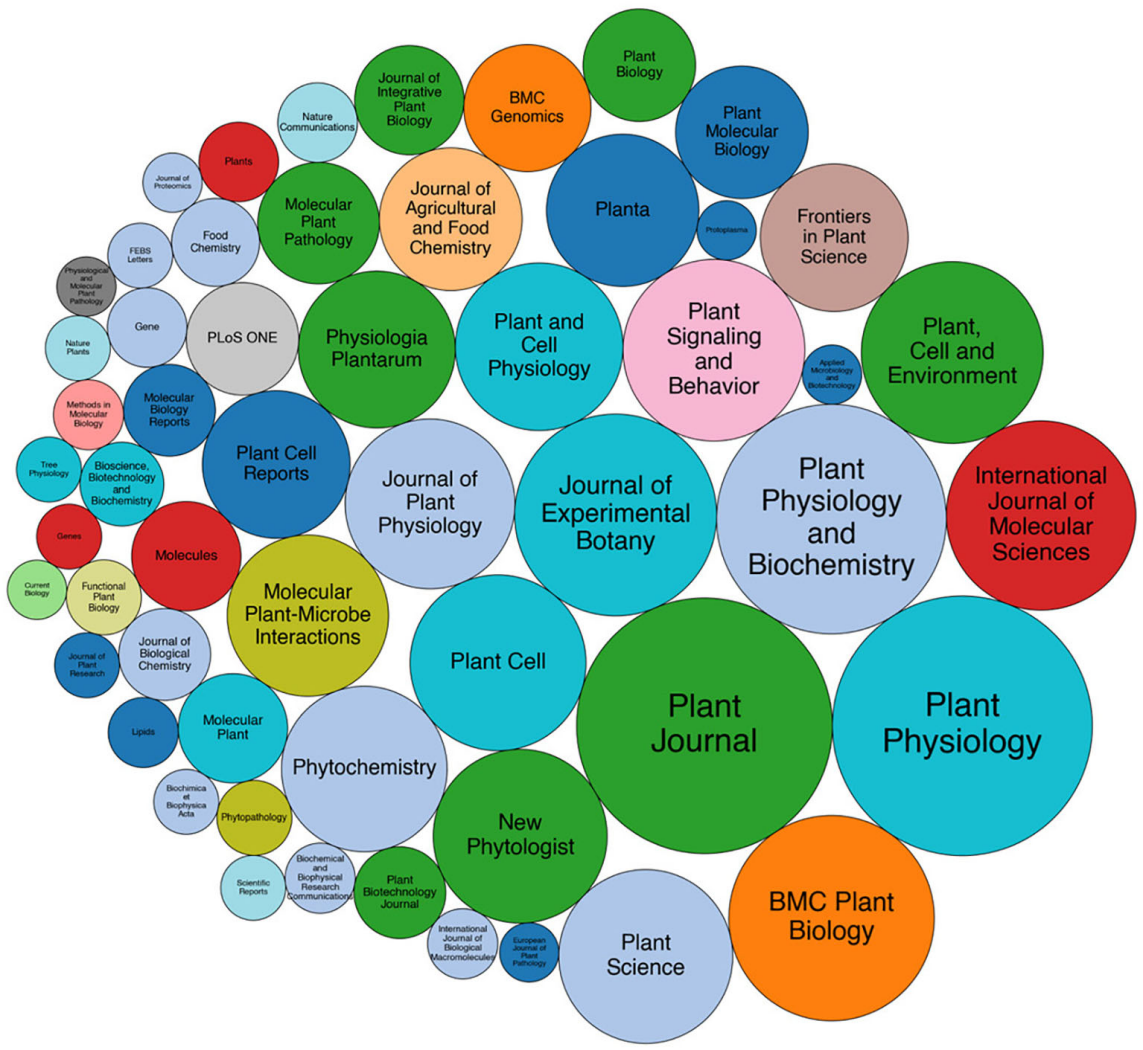
Circular tree map of all source titles identified during the research process. Circle-packing visualization was generated using the *circlify* and *matplotlib* Python libraries. Journals with fewer than five publications were excluded to enhance clarity. Each circle’s size was scaled according to the number of articles per journal, and colors were assigned by publisher.

**Figure 3:**
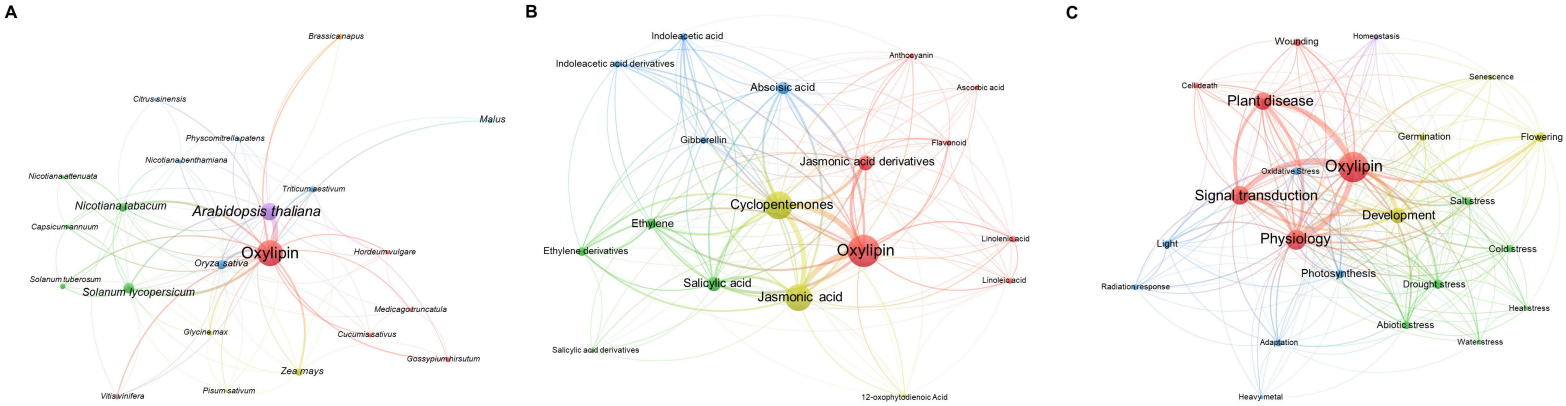
Scientific mapping of strictly linked networks for oxylipin as a keyword and **(A)** plant species, **(B)** signaling molecules, and **(C)** physiological functions, based on papers identified during the research process. The maps were elaborated using VOSviewer (v.1.6.20).

Oxylipins play a pivotal role in the plant-environment interaction (Knieper et al., 2023). A literature survey was conducted to assess the scientific interest in plant oxylipins, revealing a steady increase over the last two decades (**Figure 1**). Indeed, literature search using the Ovid and Scopus databases on the topic “plant oxylipins” revealed a total number of 1,631 research studies between 2000 and 2024 with the peak of papers published in 2024, after applying some inclusion/exclusion criteria, i.e., excluding duplicates, including only research article published in English. Six-hundreds and sixty-five papers focused on the involvement of plant oxylipins in biotic stress, 296 on abiotic, 90 on both stresses, and 580 on other physiological processes, like germination and development. A treemap was also drawn pointing out that most papers were published on Plant Physiology (96), following by Plant Journal (93) and Plant Physiology and Biochemistry (75) (**Figure 2**). By scrutinizing the co-occurrence of keywords major links were revealed between oxylipins and the plant species *Arabidopsis thaliana*, *Oryza sativa* and *Solanum lycopersicum* (**Figure 3A**). Moreover, oxylipins showed connections with the signaling molecules cyclopentenones, jasmonic and salycilic acid, and implications in several physiological functions, signal transduction and plant disease responses (**Figures 3B, C**, respectively). Indeed, the extremely diverse chemical nature of these molecules implies their involvement in several roles. It’s well known that many oxylipins are powerful regulators of plant growth, development and interactions with biotic and abiotic stressors (Sugimoto et al., 2022). The oxylipin signaling occurs through a genetically defined signal network that is linked to several additional phytohormones, like salicylic acid, ethylene, and auxin. Therefore, jasmonates and cyclopentenone lipids, such as oxo-phytodienoic acid (OPDA and dinor OPDA), can activate or repress gene expression through the electrophilic activities of the cyclopentenone ring (Sugimoto et al., 2022).

**Figure 4:**
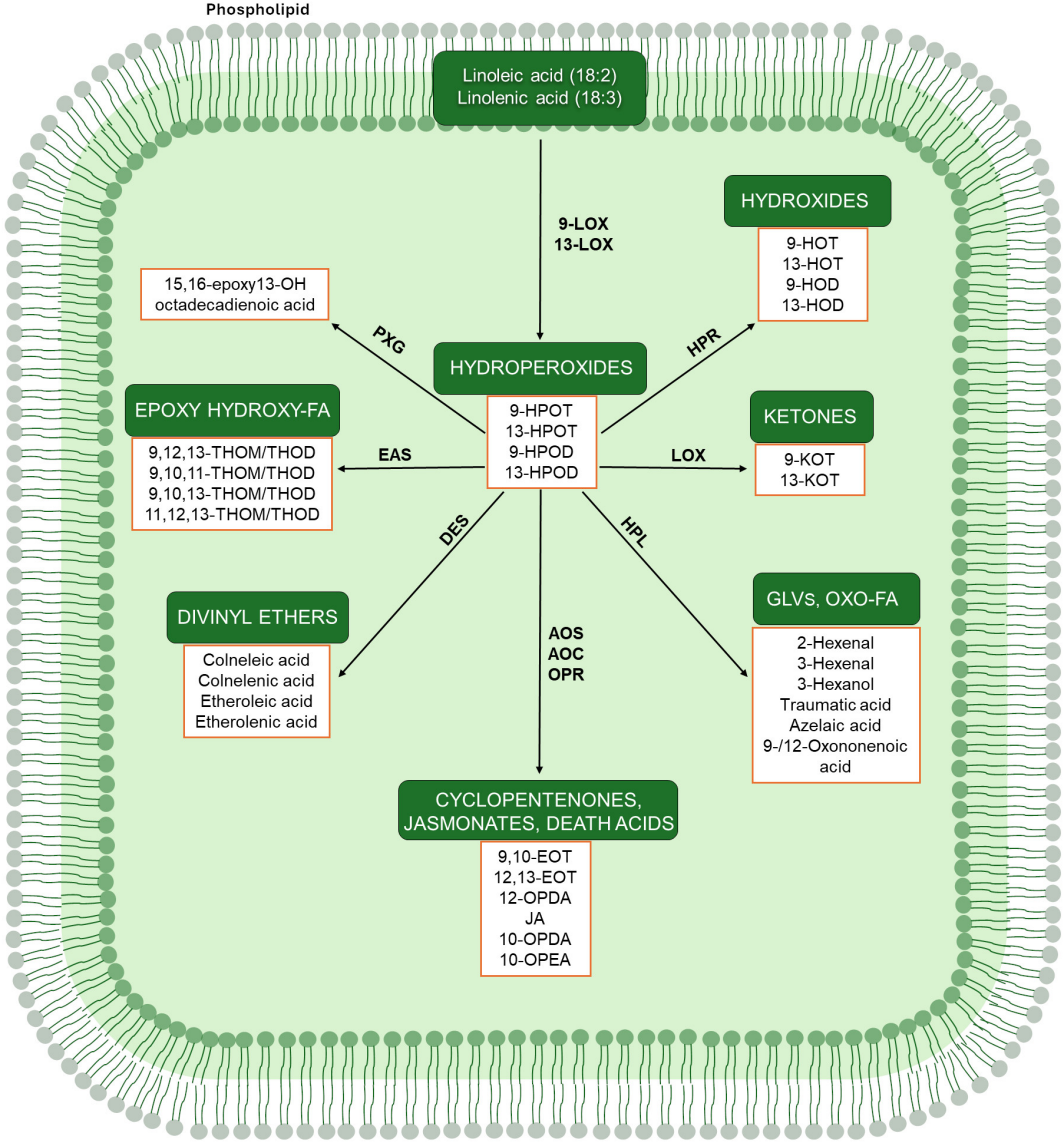
Plant oxylipin biosynthesis. Abbreviations: LOX, lipoxygenase; HPR, hydroperoxide reductase; HPL, hydroperoxide lyase; AOS, allene oxide synthase; AOC, allene oxide cyclase; OPR, 12-oxy phytodienoate reductase; DES, divinyl ether synthase; EAS, epoxy alcohol synthase; PXG, peroxygenase; GLVs, green leaf volatiles; FA, fatty acids; HPOT, hydroperoxy octadecatrienoic acid; HPOD, hydroperoxy octadecadienoic acid; HOT, hydroxy octadecatrienoic acid; HOD, hydroxy octadecadienoic acid; KOT, keto-octadecatrienoic acid; EOT, epoxy decaoctatrienoic acid; OPDA, oxo-phytodienoic acid; JA, jasmonic acid; OPEA, oxo-phytoenoic acid; THOM, trihydroxy-octadecenoic acid; THOD, trihydroxy-octadecadienoic acid.

The aim of this review is first to explore the phylogenetic relationships of the main group of enzymes participating in the biosynthesis of oxylipins considering five plant species, *Arabidopsis thaliana*, *Oryza sativa*, *Zea mays*, *Solanum lycopersicum*, and *Vitis vinifera*. The selection is based on their relevance in agriculture and availability of oxylipin-related gene data. The examined enzymes include the group of lipoxygenases (LOXs), allene oxide synthase (AOS) and cyclase (AOC), hydroperoxide lyase (HPL), divinyl ether synthase (DES), epoxy alcohol synthase (EAS), and peroxygenase (PXG) (**Figure 4**). Moreover, the contribution of oxylipins to environmental adaptation focusing on abiotic stressors, like heat, drought and waterlogging, and their role in mycotoxin production will be discussed. Lastly, the most recent genome editing interventions on the enzymes of the oxylipin pathway will be described.

## 2 Major enzymes of the plant oxylipin pathway

### 2.1 Lipoxygenases

Plant oxylipins are signaling metabolites deriving from the oxidative conversion of polyunsaturated fatty acids (PUFAs) such as linoleic (C18:2) and linolenic (C18:3) as shown in **Figure 4** (Porta and Rocha-Sosa, 2002). In 16:3 angiosperms belonging to *Brassicaceae* family a further substrate is represented by C16:3 (Kuźniak and Gajewska, 2024). Lipoxygenases (LOXs) are the first enzymes that catalyze the enzymatic oxidation of PUFAs producing fatty acid hydroperoxides. According to regiospecificity, plant LOXs are categorized into two main subfamilies, 9- and 13-LOXs, respectively. In a few instances, a mixed regiospecificity (9/13-LOX or 13/9-LOX) can be found based on the compounds principally synthetized (Viswanath et al., 2020).

In the 9-LOX pathway, C18:2 and C18:3 hydroperoxides are converted to 9-hydroperoxy octadecadienoic acid (9-HPOD) and 9-hydroperoxy octadecatrienoic acid (9-HPOT), respectively. An additional 9-LOX-produced oxylipin is the 9-keto-octadecatrienoic acid (9-KOT) (**Figure 4**). The major metabolites deriving from the conversion of PUFAs in the 13-LOX pathway are 13-HPOD, 13-HPOT and 13-KOT (**Figure 4**). Localization profiles of four maize 9-LOXs (ZmLOX2, ZmLOX4, ZmLOX6 and ZmLOX12) extended across cytoplasm, plastids and tonoplasts, suggesting compartmentation of different oxylipin production inside of the cell (Tolley et al., 2018). Moreover, subcellular localization of maize 9-LOX was mostly consistent with Arabidopsis, implying that these isoforms are conserved between monocots and dicots (Tolley et al., 2018). Similarly, three rice 9-LOXs (OsLOX1, OsLOX3 and OsLOX10) localized into the chloroplast and cytosol (Wang et al., 2008; Long et al., 2013; Wang et al., 2023a), whereas only a chloroplast localization was observed for the tomato and grapevine LOXC and LOXA isoforms, respectively (Chen et al., 2004; Pilati et al., 2024).

**Figure 5:**
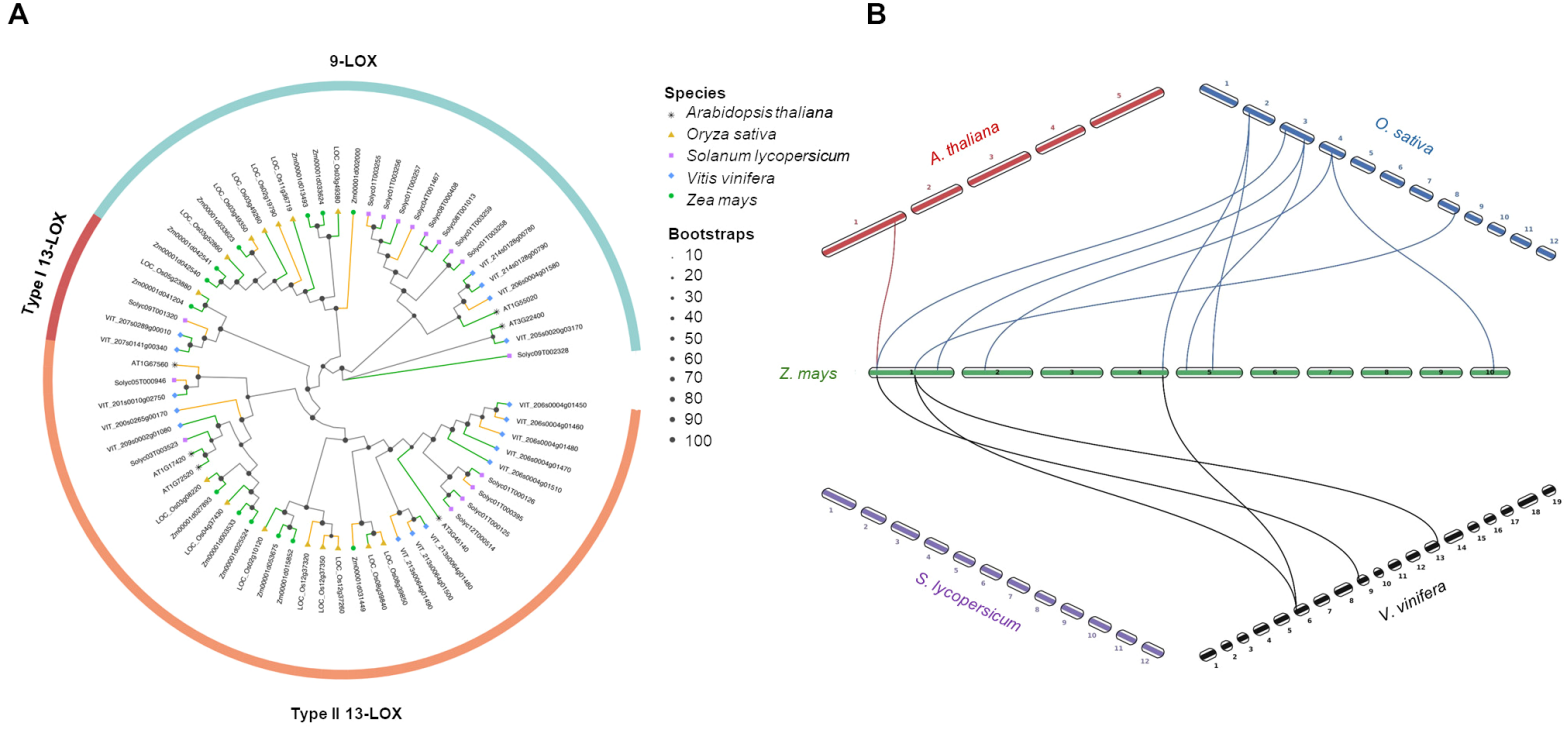
Phylogenetic tree and synteny analysis of LOX proteins in five species of plants. **(A)** In the phylogenetic tree plant species are marked with different shapes: asterisk means *Arabidopsis thaliana*, triangle means *Oryza sativa*, rectangle means *Solanum lycopersicum*, rhombus means *Vitis vinifera*, and circle means *Zea mays*. The tree was constructed by using maximum likelihood method with IQ-TREE v3.0.1 software (Wong et al., 2025) and plotted using the *ggtree* R package. Tree inference was performed using the JTT+I+G substitution model, and ultrafast bootstrap with 1000 replicates (Hoang et al., 2018). **(B)** Synteny analysis was performed between *Z. mays* and each of the other four species (*A. thaliana*, *O. sativa*, *S. lycopersicum*, and *V. vinifera*) in pairwise comparisons. Collinearity and ortholog detection were carried out using the One-Step Multiple Collinearity Scan (MCScanX) function, with default parameters (E-value of 1e^-10^ and number of BlastHits per query of 5), from the TBtools-II v2.310 software (Chen et al., 2023). The identified orthologs and their genomic coordinates were visualized using the *jcvi.graphics.karyotype* function from the JCVI Python library (Tang et al., 2024).

Based on the full-length amino acid sequences of 67 LOX proteins among five species (6 LOXs in *A. thaliana*, 15 in *O. sativa*, 13 in *Z. mays*, 16 in *S. lycopersicum*, and 17 in *V. vinifera*), a phylogenetic tree was constructed using the maximum likelihood method (**Figure 5A**). The result showed that the 67 LOX proteins across these species were classified into two subfamilies: 9- and 13-LOX. For each group, the number of LOX proteins varied. Specifically, the 13-LOX subfamily was the largest group with 40 differential members. The 13-LOX subfamily could be further clustered into two subgroups (Type I and II), in which Type II 13-LOX were universally present in all species, whereas Type I was missing in *A. thaliana*. The higher number of clade members observed in *S. lycopersicum* and *V. vinifera* probably reflects gene expansion events after cot-monocot divergence. To better understand the evolutionary mechanisms of *LOX* genes, a synteny analysis was carried out comparing *ZmLOX* with those from the other four species (**Figure 5B**). One, eight and four *ZmLOX* genes had a synteny relationship with Arabidopsis, *O. sativa* and *V. vinifera*, respectively, whereas any synteny was observed between maize and tomato.

### 2.2 CYP74 enzymes

The different pools of PUFA hydroperoxides deriving from 9- and 13-lipoxygenases are further metabolized by other enzymes located downstream in the pathway such as AOS and AOC, HPL, DES, EAS, and PXG (**Figure 4**).

#### 2.2.1 Biosynthesis of jasmonates and cyclopentenones and their mechanisms of signaling

**Figure 6:**
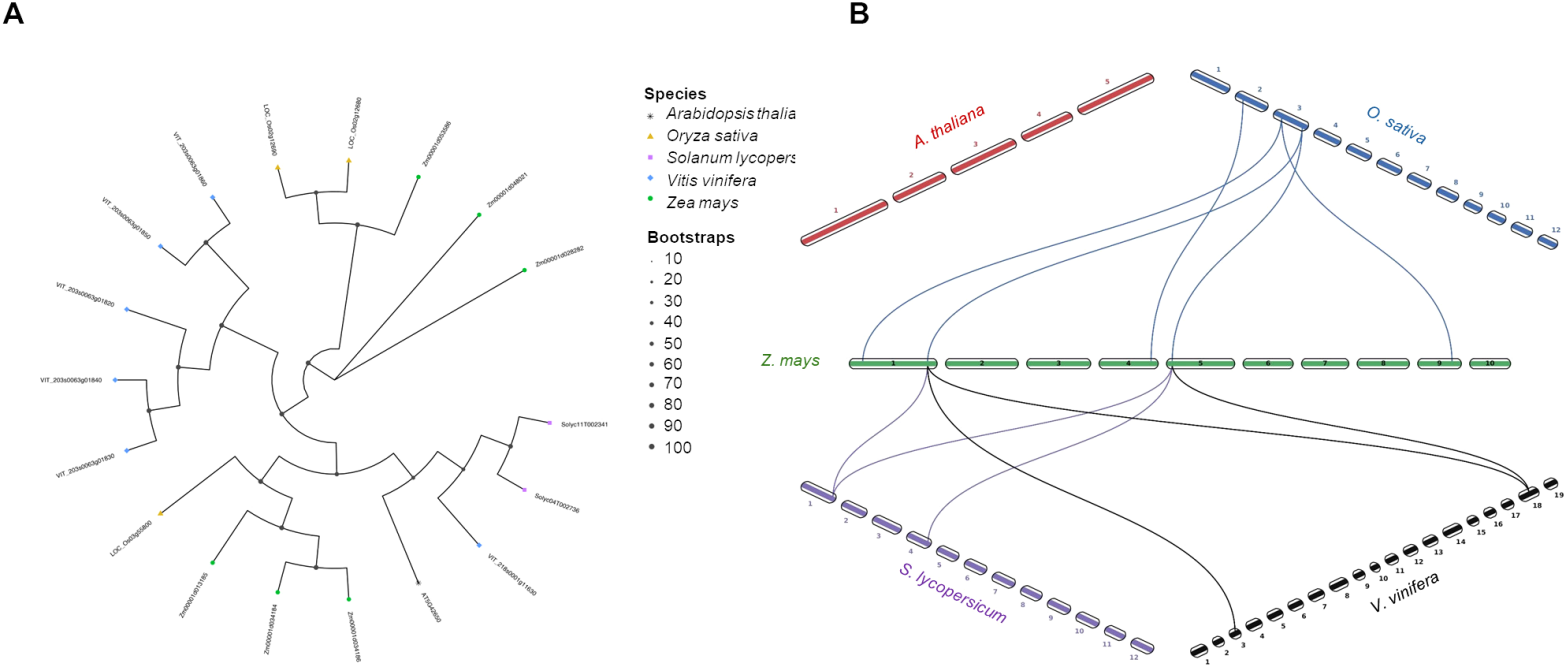
Phylogenetic tree and synteny analysis of AOS proteins in five species of plants. **(A)** In the phylogenetic tree plant species are marked with different shapes: asterisk means *Arabidopsis thaliana*, triangle means *Oryza sativa*, rectangle means *Solanum lycopersicum*, rhombus means *Vitis vinifera*, and circle means *Zea mays*. The tree was constructed by using maximum likelihood method with IQ-TREE v3.0.1 software (Wong et al., 2025) and plotted using the *ggtree* R package. Tree inference was performed using the JTT+I+G substitution model, and ultrafast bootstrap with 1000 replicates (Hoang et al., 2018). **(B)** Synteny analysis was performed between *Z. mays* and each of the other four species (*A. thaliana*, *O. sativa*, *S. lycopersicum*, and *V. vinifera*) in pairwise comparisons. Collinearity and ortholog detection were carried out using the One-Step Multiple Collinearity Scan (MCScanX) function, with default parameters (E-value of 1e^-10^ and number of BlastHits per query of 5), from the TBtools-II v2.310 software (Chen et al., 2023). The identified orthologs and their genomic coordinates were visualized using the *jcvi.graphics.karyotype* function from the JCVI Python library (Tang et al., 2024).

The biosynthesis of jasmonic acid (JA) starts in the chloroplast with the conversion of 13-hydroperoxides by 13-AOS using the substrate 13-hydroperoxy octadecatrienoic acid (HPOT) and producing the 12,13-epoxy decaoctatrienoic acid (EOT) (**Figure 4**). In contrast, the 9-AOS pathway is specific for the 9-hydroperoxide derivatives catalyzing the synthesis of 9,10-EOT in the cytosol (**Figure 4**). However, in many plant species dual substrate specificity was also detected for both 9- or 13-HPOT by AOSs (Maucher et al., 2000; Wang et al., 2008; Cho et al., 2011; Yoeun et al., 2013; Ogorodnikova et al., 2015). By analyzing B73 maize genome 6 AOS isoforms were identified (Zm00001d034186 ZmAOS1a; Zm00001d034184 ZmAOS1b; Zm00001d013185 ZmAOS1c; Zm00001d028282 ZmAOS2a; Zm00001d048021 ZmAOS2b; and Zm00001d053586 ZmAOS3). The same number of isoforms were observed in grapevine (VIT_203s0063g01860, VIT_203s0063g01850 VIT_203s0063g01840, VIT_203s0063g01820, VIT_203s0063g01830 and VIT_218s0001g11630), followed by rice (LOC_Os02g12690, LOC_Os02g12680 and LOC_Os03g55800), tomato (Solyc11T002341 and Solyc04T002736) and one isoform in Arabidopsis (AT5G42850) (**Figure 6A**). These isoforms were clustered in three clades, named AOS1, 2 and 3 (**Figure 6A**). Moreover, the synteny analysis revealed that as expected the highest number of genes in synteny with maize were found in rice, followed by a tie tomato and grapevine (**Figure 6B**).

**Figure 7:**
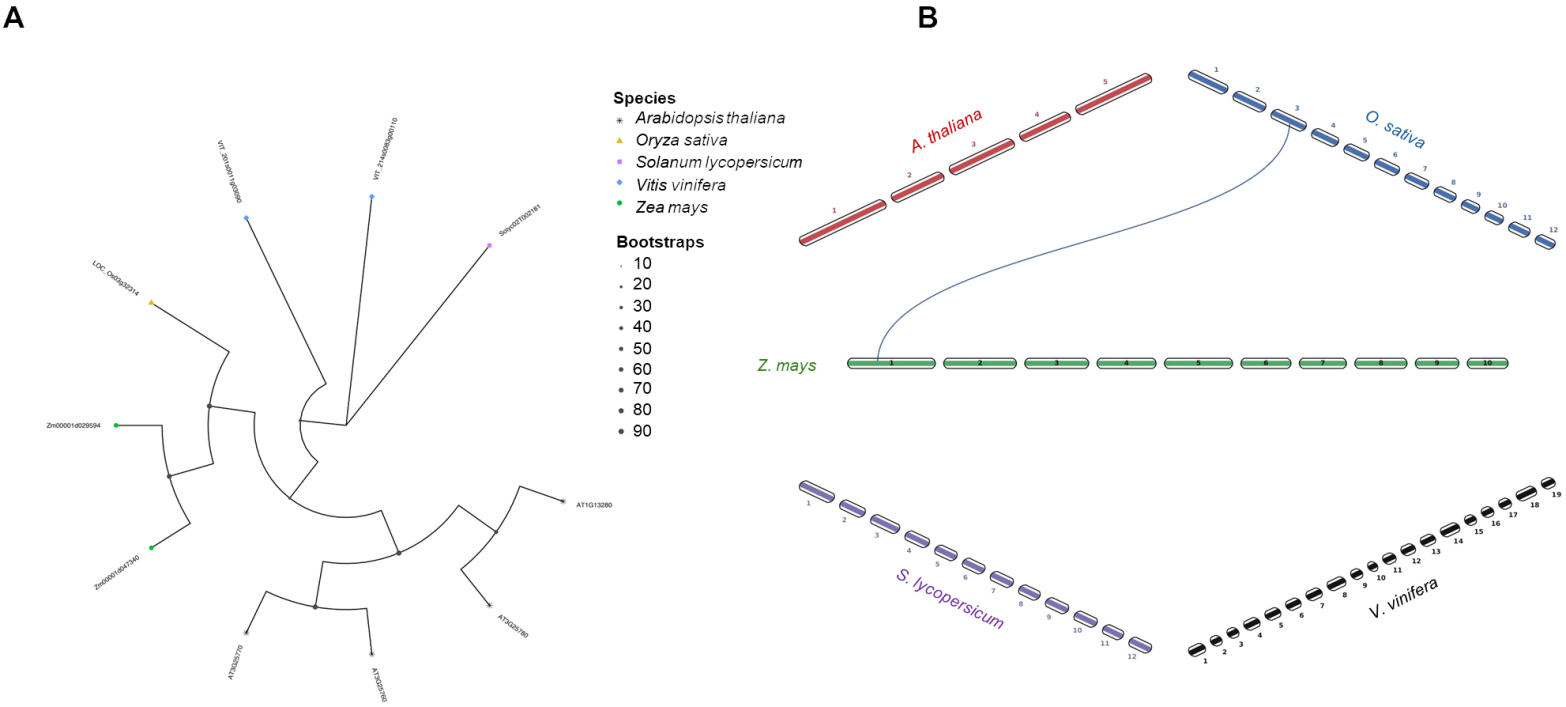
Phylogenetic tree and synteny analysis of AOC proteins in five species of plants. **(A)** In the phylogenetic tree plant species are marked with different shapes: asterisk means *Arabidopsis thaliana*, triangle means *Oryza sativa*, rectangle means *Solanum lycopersicum*, rhombus means *Vitis vinifera*, and circle means *Zea mays*. The tree was constructed by using maximum likelihood method with IQ-TREE v3.0.1 software (Wong et al., 2025) and plotted using the *ggtree* R package. Tree inference was performed using the JTT+I+G substitution model, and ultrafast bootstrap with 1000 replicates (Hoang et al., 2018). **(B)** Synteny analysis was performed between *Z. mays* and each of the other four species (*A. thaliana*, *O. sativa*, *S. lycopersicum*, and *V. vinifera*) in pairwise comparisons. Collinearity and ortholog detection were carried out using the One-Step Multiple Collinearity Scan (MCScanX) function, with default parameters (E-value of 1e^-10^ and number of BlastHits per query of 5), from the TBtools-II v2.310 software (Chen et al., 2023). The identified orthologs and their genomic coordinates were visualized using the *jcvi.graphics.karyotype* function from the JCVI Python library (Tang et al., 2024).

A tight physical association between AOS and AOC was observed at the level of the grana thylakoids in potato, even though AOS was more strongly bound to the membrane compared to AOC (Farmaki et al., 2007). Indeed, in presence of AOC the unstable allene oxide 12,13-EOT is further converted into 12-OPDA. On the other hand, in the 9-AOS pathway, 9,10-EOT undergoes to a spontaneous cyclization process bringing to the formation of 10-oxo-phytoenoic acid (OPEA) and 10-OPDA, the later known as “death acids” due to their cytotoxic activity (Christensen et al., 2015) (**Figure 4**). Four AOC isoforms are encoded by Arabidopsis genome, two for each species by *Z. mays* and *V. vinifera*, and one for each species by *O. sativa* and *S. lycopersicum* (**Figure 7A**). A closer cluster was shown for the two maize AOC1 (Zm00001d029594) and 2 (Zm00001d047340) isoforms with the only rice AOC isoform (LOC_Os03g32314), whereas the four AOCs of Arabidopsis (AT3G25770, AT3G25760, AT3G25780, and AT1G13280) clustered separately, in line with synteny results (**Figure 7A, B**). Similarly, tomato (Solyc02T002181) and grapevine AOCs (VIT_201s0011g03090 and VIT_214s0083g00110) were clustered together (**Figure 7A, B**).

It is worth highlighting that allene oxides produced by 9- and 13-AOS pathways can be also converted in 9- and 13-ketols, respectively. These included 9-hydroxy-10-oxo-12(Z)-octadecenoic acid (9,10-KOMA), 9-hydroxy-10-oxo-12(Z),15(Z)-octadecadienoic acid (9,10-KODA), 13-hydroxy-10-oxo-11(E)-octadecenoic acid (13,10-KOMA), and 13-hydroxy-10-oxo-11(E),15(Z)-octadecadienoic acid (13,10-KODA), 9,12-KOMA, 9,12-KODA, 13,12-KOMA, and 13,12-KODA. These molecules were recently described as potent signals regulating several physiological processes in plants (Berg-Falloure and Kolomiets, 2023).

**Figure 8:**
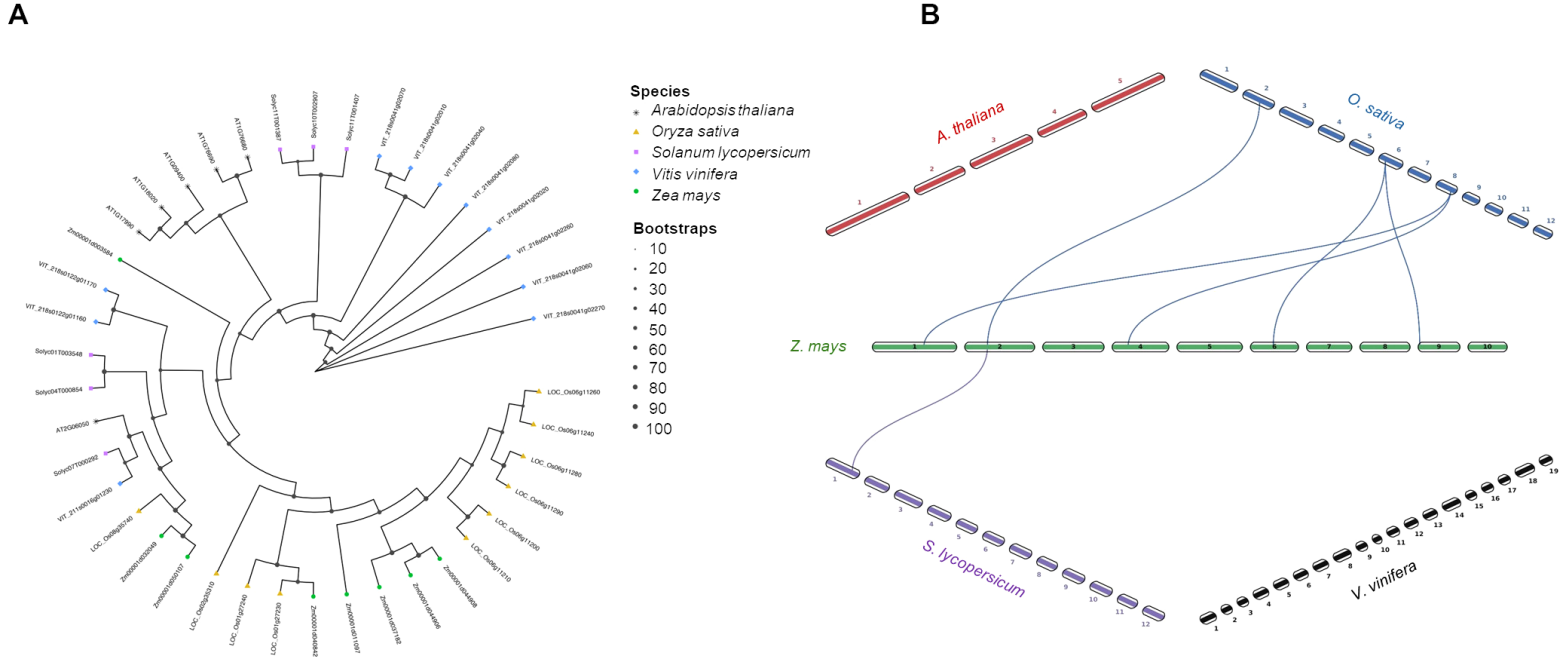
Phylogenetic tree and synteny analysis of OPR proteins in five species of plants. **(A)** In the phylogenetic tree plant species are marked with different shapes: asterisk means *Arabidopsis thaliana*, triangle means *Oryza sativa*, rectangle means *Solanum lycopersicum*, rhombus means *Vitis vinifera*, and circle means *Zea mays*. The tree was constructed by using maximum likelihood method with IQ-TREE v3.0.1 software (Wong et al., 2025) and plotted using the *ggtree* R package. Tree inference was performed using the JTT+I+G substitution model, and ultrafast bootstrap with 1000 replicates (Hoang et al., 2018). **(B)** Synteny analysis was performed between *Z. mays* and each of the other four species (*A. thaliana*, *O. sativa*, *S. lycopersicum*, and *V. vinifera*) in pairwise comparisons. Collinearity and ortholog detection were carried out using the One-Step Multiple Collinearity Scan (MCScanX) function, with default parameters (E-value of 1e^-10^ and number of BlastHits per query of 5), from the TBtools-II v2.310 software (Chen et al., 2023). The identified orthologs and their genomic coordinates were visualized using the *jcvi.graphics.karyotype* function from the JCVI Python library (Tang et al., 2024).

Following transport into peroxisomes, 12-OPDA is reduced by OPDA reductases (OPR) into the cyclopentanone OPC-8:0 (8-[3-oxo-2-cis-[(Z)-2-pentenylcyclopentyl]octanoic acid). The transport is mediated by the ABC ATP-binding cassette (ABC) transporter COMATOSE in the Arabidopsis leaves, although further import mechanisms as passive transport through peroxisome membranes could be considered (Theodoulou et al., 2005). Eleven OPR isoforms were counted in the grapevine genome, ten in rice, eight in maize, six each in tomato and Arabidopsis (**Figure 8A**). Grapevine, tomato and Arabidopsis OPR proteins tended to cluster alone with some exception, whereas maize and rice together, as also highlighted in the synteny graph (**Figure 8B**). In a previous work, the redundancy of OPR isoforms was described in maize. More in detail, maize OPR7 and 8 share about 95% of sequence identity (Borrego and Kolomiets, 2016) and only *opr7opr8* double mutants displayed reduced JA accumulation (Yan et al., 2012). Similarly, Arabidopsis *opr3* mutants still produced some JA quantities, significantly lower compared to wild-type plants, due to the presence of OPR1 and 2 isoforms (Stintzi and Browse, 2000).

**Figure 9:**
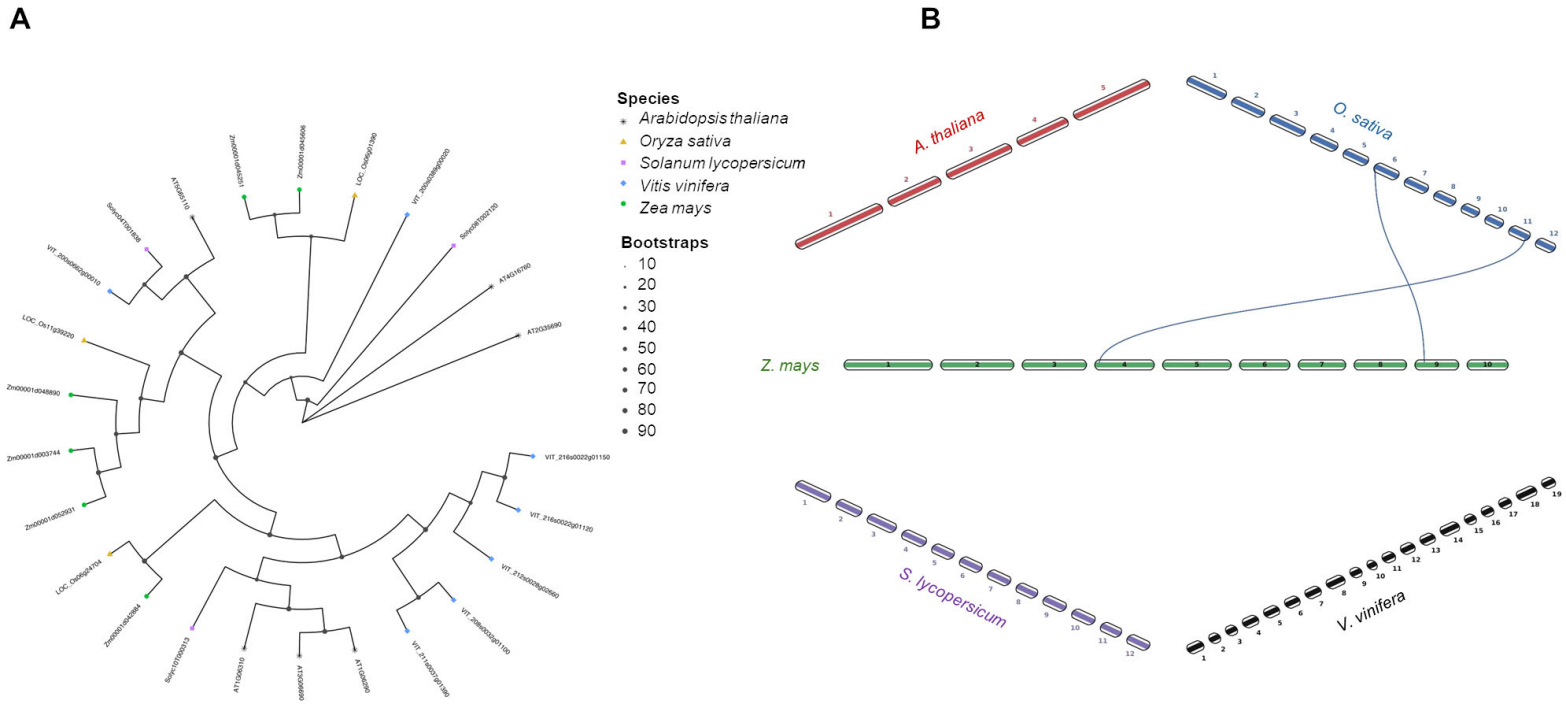
Phylogenetic tree and synteny analysis of ACX proteins in five species of plants. **(A)** In the phylogenetic tree plant species are marked with different shapes: asterisk means *Arabidopsis thaliana*, triangle means *Oryza sativa*, rectangle means *Solanum lycopersicum*, rhombus means *Vitis vinifera*, and circle means *Zea mays*. The tree was constructed by using maximum likelihood method with IQ-TREE v3.0.1 software (Wong et al., 2025) and plotted using the *ggtree* R package. Tree inference was performed using the JTT+I+G substitution model, and ultrafast bootstrap with 1000 replicates (Hoang et al., 2018). **(B)** Synteny analysis was performed between *Z. mays* and each of the other four species (*A. thaliana*, *O. sativa*, *S. lycopersicum*, and *V. vinifera*) in pairwise comparisons. Collinearity and ortholog detection were carried out using the One-Step Multiple Collinearity Scan (MCScanX) function, with default parameters (E-value of 1e^-10^ and number of BlastHits per query of 5), from the TBtools-II v2.310 software (Chen et al., 2023). The identified orthologs and their genomic coordinates were visualized using the *jcvi.graphics.karyotype* function from the JCVI Python library (Tang et al., 2024).

Subsequent β-oxidation steps shorten the extended carbon side chain of OPC:8 to form JA. The first enzyme involved in this process is acyl-CoA oxidase (ACX) that catalyzes the oxidation of fatty acid acyl-CoA to 2-trans-olefin-CoA. Several ACX isoenzymes are present in plants varying in sizes and subunit composition. Based on the carbon chain length they recognize in catalytic reactions three categories were described: long-, medium- and short-chain ACX (He et al., 2025). Phylogenetic analysis of the amino acid sequences of ACX family genes from Arabidopsis, maize, rice, tomato and grapevine revealed that the ACX family genes can be divided into four subfamilies (**Figure 9A**). The six ZmACXs (Zm00001d045606, Zm00001d045251, Zm00001d048890, Zm00001d003744, Zm00001d052931, and Zm00001d042884) clustered only with OsACXs (LOC_Os06g01390 and LOC_Os06g24704), in line with synteny plot (**Figure 9B**), whereas Arabidopsis, tomato and grapevine grouped separately (**Figure 9A**). Not all ACX isoforms are involved in JA biosynthesis, for instance in Arabidopsis and rice, only AtACX1, AtACX5 and OsACX1 take part in this process (Kim et al., 2007; Schilmiller et al., 2007). Similarly, tomato SlACX1 and tea tree CsACX1 and CsACX3 were exclusively found related to JA synthesis (Li et al., 2005; Chen et al., 2019).

**Figure 10:**
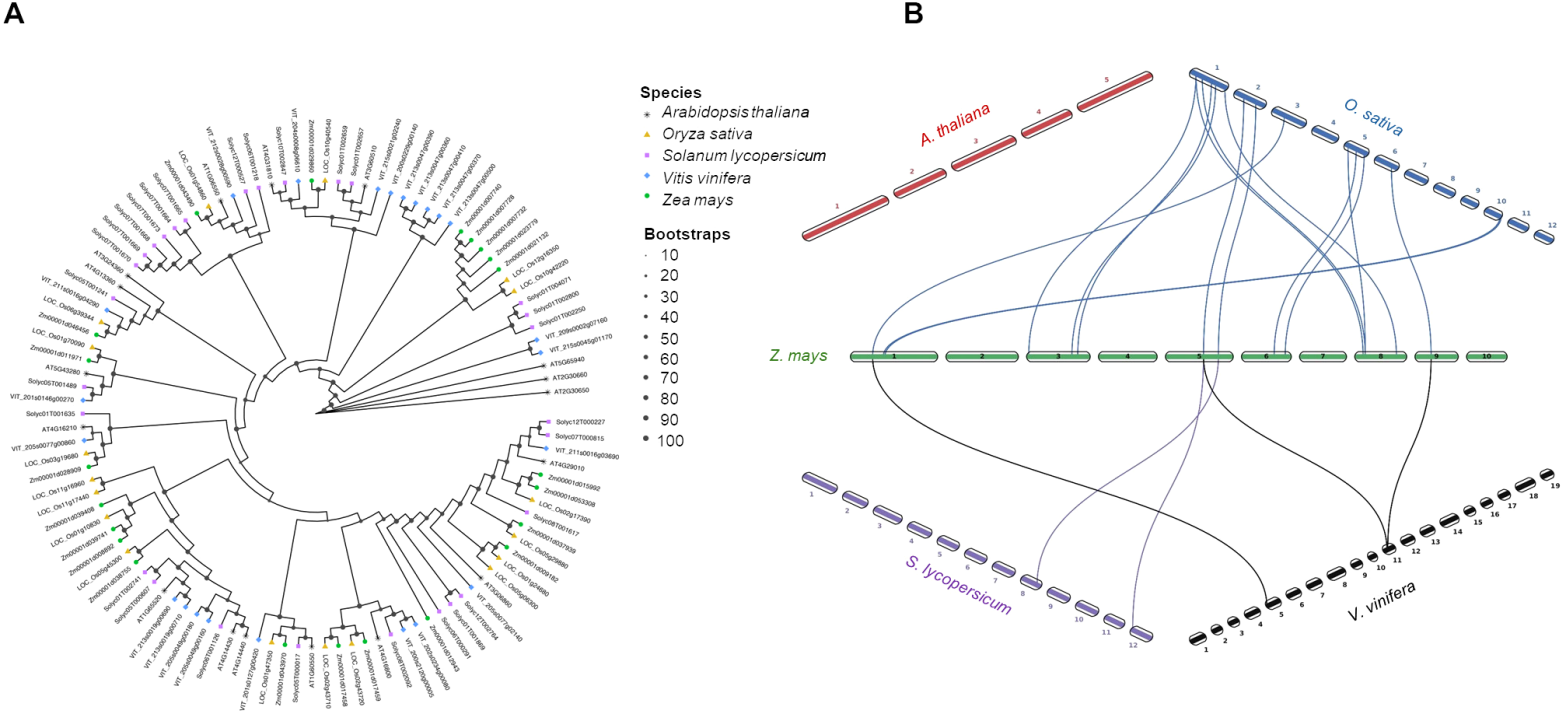
Phylogenetic tree and synteny analysis of MFP proteins in five species of plants. **(A)** In the phylogenetic tree plant species are marked with different shapes: asterisk means *Arabidopsis thaliana*, triangle means *Oryza sativa*, rectangle means *Solanum lycopersicum*, rhombus means *Vitis vinifera*, and circle means *Zea mays*. The tree was constructed by using maximum likelihood method with IQ-TREE v3.0.1 software (Wong et al., 2025) and plotted using the *ggtree* R package. Tree inference was performed using the JTT+I+G substitution model, and ultrafast bootstrap with 1000 replicates (Hoang et al., 2018). **(B)** Synteny analysis was performed between *Z. mays* and each of the other four species (*A. thaliana*, *O. sativa*, *S. lycopersicum*, and *V. vinifera*) in pairwise comparisons. Collinearity and ortholog detection were carried out using the One-Step Multiple Collinearity Scan (MCScanX) function, with default parameters (E-value of 1e^-10^ and number of BlastHits per query of 5), from the TBtools-II v2.310 software (Chen et al., 2023). The identified orthologs and their genomic coordinates were visualized using the *jcvi.graphics.karyotype* function from the JCVI Python library (Tang et al., 2024).

The multifunctional proteins (MFPs) possessing 2-trans-enoyl-CoA hydratase and L-3-ketoacyl Co A thiolase (KAT) also contribute to the β-oxidation steps. A total number of 108 MFP isoforms were counted among the five examined species (17 in *A. thaliana*, 18 in *O. sativa*, 22 in *Z. mays*, 28 in *S. lycopersicum*, and 23 in *V. vinifera*) that clustered in several subfamilies (**Figure 10A**). Moreover, 15, three and two *ZmMFP* genes had a synteny relationship with *O. sativa*, *V. vinifera* and *S. lycopersicum*, respectively, whereas any synteny was observed between maize and Arabidopsis (**Figure 10B**). Once MFP and KAT have intervened, jasmonoyl-CoA is hydrolyzed by an unknown process to free JA (Borrego and Kolomiets, 2016).

**Figure 11:**
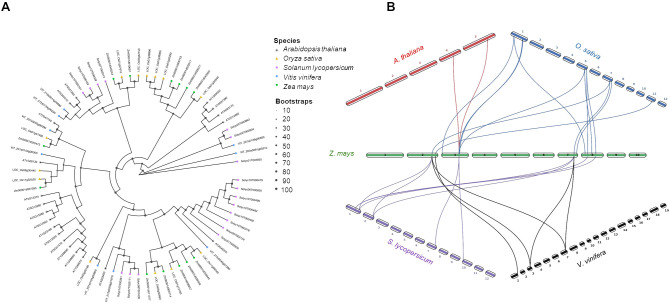
Phylogenetic tree and synteny analysis of JAR proteins in five species of plants. **(A)** In the phylogenetic tree plant species are marked with different shapes: asterisk means *Arabidopsis thaliana*, triangle means *Oryza sativa*, rectangle means *Solanum lycopersicum*, rhombus means *Vitis vinifera*, and circle means *Zea mays*. The tree was constructed by using maximum likelihood method with IQ-TREE v3.0.1 software (Wong et al., 2025) and plotted using the *ggtree* R package. Tree inference was performed using the JTT+I+G substitution model, and ultrafast bootstrap with 1000 replicates (Hoang et al., 2018). **(B)** Synteny analysis was performed between *Z. mays* and each of the other four species (*A. thaliana*, *O. sativa*, *S. lycopersicum*, and *V. vinifera*) in pairwise comparisons. Collinearity and ortholog detection were carried out using the One-Step Multiple Collinearity Scan (MCScanX) function, with default parameters (E-value of 1e^-10^ and number of BlastHits per query of 5), from the TBtools-II v2.310 software (Chen et al., 2023). The identified orthologs and their genomic coordinates were visualized using the *jcvi.graphics.karyotype* function from the JCVI Python library (Tang et al., 2024).

When released into the cytoplasm, JA can be modified by conjugation to several amino acids, methylation, decarboxylation, hydroxylation, glycosylation, sulfonation, or by more than one modification (Borrego and Kolomiets, 2016). The enzyme JASMONATE RESISTANT (JAR) is responsible for the conjugation of isoleucine with JA. Seventy-three JAR isoforms were found in Arabidopsis, rice, maize, tomato and grapevine, overall, distributed to numerous subfamilies (**Figure 11A**). Twelve *ZmJAR* genes were in synteny with rice, two with Arabidopsis, seven with tomato and five with grapevine (**Figure 11B**). The functional characterization of JAR isoforms was carried out only in a few plant species, like Arabidopsis (Staswick and Tiryaki, 2004), rice (Wakuta et al., 2011) and wheat (Tuan et al., 2022). More extensive studies will be required to better understand the role of this category of enzymes.

The signaling mechanisms in JA/JA-Ile dependent processes was extensively reviewed by Wasternack and Strnad (2018). The interaction of JA-Ile and additional JA conjugates with the CORONATINE-INSENSITIVE 1 (COI1) unit of an E3 ubiquitin ligase complex termed SCF^coi1^, where SCF means Skp/Cullin/F-box, is important in JA perception. This event is followed by subsequent recruitment of JA ZIM domain (JAZ) proteins. These proteins are repressors of JA-inducible genes. Their degradation via 26S proteasome activate MYC2 transcription factors that bind G-box motif of the promoters of JA-responsive genes (Wasternack and Strnad, 2018).

#### 2.2.2 Biosynthesis of volatile oxylipins

Hydroperoxide lyase (HPL) competes with AOS for the common substrate 13-HPOT whose cleavage results in the formation of the green leaf volatiles (GLV) 2-hexenal and traumatic acid (12-oxo-(E)-10-dodecanoic acid) (**Figure 4**). 9/13-HPLs have been also described in several plant species as melon fruit, alfalfa, almond and rice (Tijet et al., 2001; Mita et al., 2005; Chehab et al., 2006; De Domenico et al., 2007). Different subcellular localization was reported for HPL enzymes: 13-HPL pathway derivatives as 6-carbon aldehydes and 12-carbon oxoacids are produced in chloroplasts from 13-HPOT (Blée and Schuber, 1990). Conversely, 9-HPL contribute to the formation of two 9-carbon compounds from 9-hydroperoxides in the cytosol (Mita et al., 2005). The resulting HPL-derived aldehydes can be further isomerized or converted into alcohol, hydroxyl- or acetyl-containing derivatives. These metabolites represent a fundamental component of the aroma in fruits and green leaves contributing to the complex signaling system including plant–plant and plant–insect interactions (Sugimoto et al., 2022).

Additional members of CYP74 family are enzymes displaying 9/13 DES activity. The 13-DES enzymes catalyze the conversion of 13-hydroperoxides to etheroleic and etherolenic acid, whereas 9-DES activity produces colneleic and colnelenic acid (**Figure 4**). Unlike AOS and HPL, DES have been studied to a much lesser extent. Only nine genes encoding DES have been cloned, including four DES having specificity for 9-hydroperoxides (Itoh and Howe, 2001; Stumpe et al., 2001; Fammartino et al., 2007; Gullner et al., 2010), four for 13-hydroperoxides (Gogolev et al., 2012; Gorina et al., 2014, Gorina et al., 2016), and one 9/13-DES (Stumpe et al., 2008).

#### 2.2.3 Biosynthesis of epoxyalcohols

Other branches of the lipoxygenase cascade determine the formation of epoxy hydroxy derivatives also known as epoxyalcohols (**Figure 4**). Two different mechanisms cause the conversion of fatty acid hydroperoxides to epoxyalcohols. The first mechanism is catalyzed by PXG and other oxidoreductases through the reduction of the peroxy moiety and the epoxidation of one double bond (Blée and Joyard, 1996; Garscha and Oliw, 2009). The second mechanism involves the enzyme EAS discovered for the first time from the lancelet *Branchiostoma floridae* Hubbs in 2008 (Poulos, 2014). Despite the absence of EAS enzymes in plants, products of the EAS reaction have been found (Toporkova et al., 2024). The presence of epoxyalcohols in plants could be explained by the fact that several CYP74 enzymes previously characterized or annotated as AOSs, HPLs, or DESs have shown EAS activity (Toporkova et al., 2018).

Having outlined the enzymatic machinery underlying plant oxylipin biosynthesis, the following sections explore their mechanisms and functional roles in stress adaptation traits.

## 3 Oxylipin-mediated tolerance to abiotic stress

Severe climatic events such as extreme heat, drought, and heavy rainfall can significantly impact plants, and call for recurrent and easily evolutionary adaptation and acclimatization. In this context, oxylipins serve as stress mitigators, reducing the impact of abiotic stressors. These lipid-derived signaling molecules modulate gene expression patterns (Taki et al., 2005; Guche et al., 2022), antioxidant activity (Yuan et al., 2017), membrane stability (Savchenko et al., 2014), and cross-talk with other hormonal pathways to fine-tune stress responses (Knieper et al., 2023). The role of oxylipins in plant responses to various abiotic challenges is explored in this section (**Table 1**).

**Table 1:** The involvement of genes participating in the biosynthesis of oxylipins or their products during abiotic stress response.

Genes/Compounds	Method	Functions	Crop	References
*LOX2*	Parent genotypes	Resistance to drought stress	Barley	Du et al., 2013
*LOX6*; JA	Loss of function mutants	Resistance to drought stress	Arabidopsis	Grebner et al., 2013
*AOS*; 12-OPDA	Overexpressing mutants	Resistance to drought stress	Arabidopsis, tomato and canola	Savchenko et al., 2014
*OPR3*, *LOX6* and *JAR*; JA-Ile	Loss of function mutants	Resistance to drought stress	Arabidopsis	De Ollas et al., 2015
9,10-KODA	Parent genotypes	Resistance to drought stress	Wheat	Haque et al., 2016
JA	Loss of function mutants	Resistance to drought stress	Tomato	De Ollas et al., 2018
MetJA	Parent genotypes	Resistance to drought stress	Wheat	Allagulova et al., 2020
JA	Overexpressing mutants	Resistance to drought stress	Cassava	Li et al., 2022a
*JAR*; JA-Ile	Loss of function mutants	Resistance to drought stress	Arabidopsis	Mahmud et al., 2022
*LOX2*; JA	Loss of function mutants	Resistance to drought stress	Sea buckthorn	Yao et al., 2022
JA	Parent genotypes	Resistance to drought stress	Barley	Aliakbari et al., 2024
JA	Parent genotypes	Resistance to drought stress	Soybean	Rahman et al., 2024
MetJA	Parent genotypes	Resistance to drought stress	Rice	Samota et al., 2024
*JAZ10*	Loss of function mutants	Resistance to drought stress	Barley	Wu et al., 2024
*LOX1*-*LOX13*	Parent genotypes	Resistance to drought, cold, heat and salt stress	Maize	Li et al., 2025
12-OPDA	Parent genotypes	Resistance to heat stress	Arabidopsis	Mueller et al., 2008
*LOX1*	Parent genotypes	Resistance to cold stress	Arabidopsis	Arbona et al., 2010
*LOX9*	Parent genotypes	Resistance to cold stress	Cucumber	Yang et al., 2012
JA	Loss of function mutants	Resistance to cold stress	Arabidopsis	Hu et al., 2013
LOX	Parent genotypes	Resistance to cold and heat stress	Wheat	Kosakivska et al., 2014
JA	Loss of function mutants	Resistance to carbon dioxide	Canola	Geng et al., 2016
*LOX2*, *AOS1* and *OPR1*; 12-OPDA and JA	Loss of function mutants	Resistance to cold stress	Rice	Sharma and Laxmi, 2016
*JA* genes; JA and JA-Ile	Loss of function mutants	Resistance to heat stress	Arabidopsis	Balfagón et al., 2019
RES-oxylipins	Loss of function mutants	Resistance to cold and heat stress	Common liverwort	Monte et al., 2020
12-OPDA	Parent genotypes, loss of function and overexpressing mutants	Resistance to heat stress	Several	Liu and Park, 2021
*LOX1* and *LOX3*	Parent genotypes	Resistance to heat stress	Hard fescue	Xu et al., 2021
*JOXs* and *ST2A*	Loss of function and overexpressing mutants	Resistance to heat stress	Arabidopsis	Zhu et al., 2021
*JAZ*; JA	Loss of function and overexpressing mutants	Resistance to cold stress	Rice	Zeng et al., 2022
GLVs	Parent genotypes, loss of function and overexpressing mutants	Resistance to cold and heat stress	Several	Knieper et al., 2023
*JAR1*; JA	Parent genotypes and loss of function mutants	Resistance to cold stress	Wheat	Zhang et al., 2023
13-HLA, 12,13-ELA and JA	Overexpressing mutants	Resistance to cold stress	Tomato	Liu et al., 2024
*LOX2*	Parent genotypes	Resistance to waterlogging stress	Soybean	Chen et al., 2016
*LOXs*	Loss of function and overexpressing mutants	Resistance to waterlogging stress	Arabidopsis	Yuan et al., 2017
*SRG2* and *LOX1*; 12-OPDA	Loss of function mutants	Resistance to waterlogging and arsenic stress	Arabidopsis	Kumar et al., 2019
AOS, HPL and 12-OPDA	Parent genotypes, loss of function and overexpressing mutants	Resistance to waterlogging stress	Arabidopsis	Savchenko et al., 2019
LOX	Parent genotypes	Resistance to waterlogging stress	Maize	Hu et al., 2020
*LOX*	Overexpressing mutants	Resistance to waterlogging stress	Kiwifruit	Li et al., 2022b

AOS, allene oxide synthase; HPL, hydroperoxide lyase; JA, jasmonic acid; JA-Ile, jasmonoyl-l-isoleucine; JAR, JASMONATE RESISTANT; JAZ, jasmonate ZIM domain; JOX, JASMONATE-INDUCED OXYGENASE; LOX, lipoxygenase; MetJA, methyl jasmonate; OPR, OPDA reductase; RES, Reactive electrophiles; 12,13-ELA, 12,13-epoxy linolenic acid; 13-HLA, 13-hydroperoxy linolenic acid; 9,10-KODA, 9-hydroxy-10-oxo-12(Z),15(Z)-octadecadienoic acid; 12-OPDA, 12-oxo-phytodienoic acid.

### 3.1 Drought stress

Drought stress represents a major challenge affecting plant development and crop yield, globally. It induces complex physiological and molecular responses aimed at minimizing cellular damage and ensuring survival. A crucial role in managing plant water demand is played by lipids, in particular the dehydration process triggers the production of phosphatidic acid, inositol phosphates, sphingolipids and oxylipins as well (Sharma et al., 2023) (**Table 1**). The connection between oxylipin levels and drought tolerance was reported in Arabidopsis defective mutants in LOX6, which resulted impaired in stress-induced jasmonate synthesis and were more susceptible to drought (Grebner et al., 2013). In the same species, JASMONATE RESISTANT overexpressing lines showed increased jasmonoyl-l-isoleucine (JA-Ile) production, and under drought stress conditions displayed reduced wilting and recovered better from desiccation than the wild type plants (Mahmud et al., 2022). Recently, the expression profiling of thirteen *ZmLOX* genes in response to different abiotic stresses revealed a high responsiveness for six genes under drought condition, notably *ZmLOX2* was induced up to 70-fold at 24 h after treatment (Li et al., 2025). Similarly, a strong up-regulation was reported in rice for *OsLOX2* (Du et al., 2013). Research has shown a close interaction between JA and abscisic acid (ABA), the primary hormone responsible for drought tolerance (Riemann et al., 2015; Wu et al., 2024). The two hormones can act synergistically to regulate stomatal closure, reducing water loss due to transpiration (Riemann et al., 2015). Savchenko et al. (2014) found that 12-OPDA was the functional convergence point between oxylipin and ABA biosynthesis pathways. By employing *A. thaliana* ecotypes, they observed an accumulation of both 12-OPDA and JA after wounding along with low levels of ABA, whereas drought treatment determined an enhanced accumulation of only 12-OPDA and ABA. Moreover, Arabidopsis mutant plants producing higher 12-OPDA levels showed improved drought tolerance. The exogenous application of ABA and 12-OPDA, whether individually or combined, promoted stomatal closure in tomato and canola ABA and AOS biosynthetic mutants (Savchenko et al., 2014). Interestingly, seed priming with methyl jasmonate (MetJA) mitigated drought stress-induced oxidative burst in rice plants. As a consequence, this improved the accumulation of carotenoids, ABA, proline and the activities of ascorbate peroxidase, superoxide dismutase, NADPH oxidase, and catalase along the up-regulation of drought-responsive genes (Samota et al., 2024). Likewise, JA priming of soybean plants enhanced plant biomass, photosynthetic efficiency and leaf relative water content under single and combined heat and drought conditions, supporting the antioxidant defense system (Rahman et al., 2024). In addition, wheat seeds imbibed with 9,10-KODA showed improved germination, yield and growth during drought stress (Haque et al., 2016). Overall, these studies indicate that oxylipin signaling, particularly via 12-OPDA and jasmonates, plays a central role in coordinating drought-induced protective responses.

### 3.2 Thermotolerance

Collectively, the above evidence suggests that oxylipins contribute to both basal and acquired thermotolerance by modulating JA levels and stress-related gene expression. Lipoxygenases efficiently act as molecular markers for monitoring thermal stress in plants (Singh et al., 2022) (**Table 1**). The expression of *LOX* genes was previously reported under low-temperature conditions in Arabidopsis (*LOX1*; Arbona et al., 2010), maize (*LOX1*-*LOX4*, and *LOX6*-*LOX11*; Li et al., 2025), and cucumber (*LOX9*; Yang et al., 2012). Several additional genes of jasmonate biosynthesis were up-regulated under cold stress in rice, including *AOS1* and *OPR1*, leading to an accumulation of JA and 12-OPDA (Sharma and Laxmi, 2016). In this regard, Hu et al. (2013) found that JA regulates the cbf expression-C-repeat binding factor/DRE binding factor1 transcriptional machinery involved in Arabidopsis freezing tolerance. An accumulation of the JA biosynthesis substrates 13-hydroperoxy linolenic acid and 12,13-epoxy linolenic acid was observed in tomato plants overexpressing *Ammopiptanthus nanus GolS1*, a gene involved in the galactinol synthesis (Liu et al., 2024). In addition, exogenous galactinol treatment determined a JA content increase as well as a cold tolerance enhancement (Liu et al., 2024). Wheat plants overexpressing the plastid-lipid-associated protein 6, chloroplastic-like (*TaPAP6L*) showed increased JA levels and improved cold tolerance (Zhang et al., 2023). Transcriptome sequencing of these plants revealed 27 significantly up-regulated genes involved in the synthesis of linolenic acid, whereas three JA-amino synthetases, JAR1s, responsible for JA degradation into JA-Ile, were significantly down-regulated (Zhang et al., 2023). Overexpression and RNAi of rice *OsClo5*, a caleosin gene involved in the maintenance of lipid droplet structure and in signal transduction, decreased and increased cold tolerance, respectively (Zeng et al., 2022). In the overexpressing plants the inhibition of JA synthesis was observed along the up-regulation of *JAZ* genes, known to be inhibitor of the JA signal transduction pathway (Zeng et al., 2022).

Cold temperatures also cause condensation and deposition on plant surfaces of GLV triggering pollination issues and increasing biotic stress severity (Knieper et al., 2023). On the other hand, climate change and global temperature rising determined an increment of GLVs by 10% in the last 30 years (Knieper et al., 2023). Reactive electrophile oxylipins (RES-oxylipins), also referred to as reactive carbonyl species, play a key role in stress defense towards thermotolerance too (Monte et al., 2020). Heat stress acclimation is mediated by heat shock proteins, whose synthesis is induced by 12-OPDA, phytoprostanes, and malondialdehyde (Mueller et al., 2008; Liu and Park, 2021). Both high (+40°C) and low (+4°C) temperatures supported LOX activity in wheat seedlings (Kosakivska et al., 2014). Comparative transcriptomic profiles of two genotypes of hard fescue (*Festuca trachyphylla*) contrasting in heat tolerance revealed as central hub genes *LOX1* and *LOX3*, together with phenylalanine ammonia lyase and dhurrin, found up-regulated only in the heat-tolerant line (Xu et al., 2021). Elevated temperatures combined with high light intensities often occurred with enhanced accumulation of JA and JA-Ile, the up-regulation of many JA-associated genes and distinctive structural changes to chloroplasts in Arabidopsis (Balfagón et al., 2019). Warm temperature also led to an increased expression of *JASMONATE-INDUCED OXYGENASES* (*JOXs*) and *ST2A*, genes controlling JA catabolism, reducing the level of bioactive jasmonates in Arabidopsis. In the end this resulted in more JAZ proteins, which facilitated organ elongation and enhanced cooling capacity (Zhu et al., 2021). In addition, Geng et al. (2016) found that JA signaling mediated stomatal closure induced by treatment with elevated concentrations of carbon dioxide, the primary driver of climate change, raising reactive oxygen species production in *Brassica napus* guard cells.

### 3.3 Waterlogging stress

A further consequence of climate change is flooding stress that negatively affects plant growth and development (Li et al., 2022b). Water submergence of root tissues causes a rapid decline in oxygen availability, reducing aerobic respiration, determining nutrient deficiency, oxidative stress, and toxic compound accumulation, and developing chlorosis, wilting, and rotting symptoms (Phukan et al., 2016). The contribution of oxylipin metabolism to flooding stress in plants was previously described (**Table 1**). Transcriptome analysis of the roots of KR5, a kiwifruit tolerant genotype, revealed the involvement of ‘fatty acid metabolism and biosynthesis’ and ‘alpha-linolenic acid metabolism’ pathways 72 h after waterlogging stress with eleven genes found highly promoted, one of which encoding a *lipoxygenase* (Li et al., 2022b). *Lipoxygenase 2* was specifically up-regulated under flooding stress in *Glycine max* (Chen et al., 2016). Similarly, an increased expression of several *LOXs* was observed in Arabidopsis during post-submergence reoxygenation (Yuan et al., 2017). Moreover, LOX activity was significantly induced under waterlogging stress in maize (Hu et al., 2020). Allene oxide synthase (AOS) and hydroperoxide lyase (HPL) branches can also contribute to the adaptation process under waterlogging conditions (Savchenko et al., 2019). Targeted metabolomics of Arabidopsis genotypes grown under waterlogged conditions increased levels of AOS- and HPL-derived metabolites, in particular of 12-OPDA. In addition, the survival rates of plants under submergence stress ranged from 55 to 77% when only one or both pathways were active, respectively. Conversely, survival rate fell to about 30% in double AOS and HPL knockout mutants (Savchenko et al., 2019). The strong accumulation of 12-OPDA was also observed in Arabidopsis leaves when arsenic toxicity was combined with hypoxia. Therefore, elevated JA marker transcript amount, *SRG2* and *LOX1*, were detected in stressed roots (Kumar et al., 2019). It was reported that the expression of redox metabolism‐related genes like *OXI1*, primarily associated to oxidative burst signal transduction pathway in Arabidopsis, can be influenced by OPDA (Taki et al., 2005).

Most of the studies regarding thermotolerance and waterlogging stress described so far have been carried out under controlled conditions such as growth chambers or greenhouses. More practical field-based research will be necessary to validate and confirm the involvement of genes participating in the biosynthesis of oxylipins or their products in broad or species-specific responses to these stresses.

## 4 Oxylipin-mediated tolerance to mycotoxin contamination

How climate change affects mycotoxin contamination in edible crops was extensively reviewed by Casu et al. (2024). Plant oxylipins act as signals to modulate fungal developmental processes, including sporogenesis and biosynthesis of mycotoxins (Beccaccioli et al., 2022) (**Table 2**). Strong changes in oxylipin, sphingolipid, phospholipid, phytoceramide and amadori-glycated glycerophosphoethanolamine accumulation in response to fumonisin contamination and its source *Fusarium verticillioides* were previously reported in different maize lines and hybrids under open field conditions (Giorni et al., 2015; Righetti et al., 2021; Carbonell-Rozas et al., 2025). It was observed that *ZmLOX4* gene loss of function mutation compromised resistance to *F. verticillioides* and fumonisin content in maize seedlings (Lanubile et al., 2021b) and ears (Guche et al., 2022) and altered *ZmLOX* gene expression as well as LOX enzymatic activity. Moreover, in Guche et al. (2022) work *lox4* mutants were also highly susceptible to *Aspergillus flavus* and aflatoxin contamination. In contrast, *ZmLOX4* overexpressing lines were significantly less inclined to the fungus and fumonisin contamination showing a stronger induction of 9- and 13-LOX genes along with an increased production of multiple 9-oxylipins and JA amounts (Ottaviani et al., 2026). The 9-lipoxygenase ZmLOX12 is also required to limit *F. verticillioides* infection in maize, indeed, *lox12* loss of function mutants showed a wide fungal colonization of mesocotyls, stalks and kernels, a great amount of fumonisins and reduced levels of 12-OPDA, JA and JA-Ile and expression of JA-biosynthetic genes (Christensen et al., 2014). Contrasting findings were instead reported for the gene *ZmLOX3* whose knock-out mutants were found more resistant to *F. verticillioides* colonization with a striking reduction of fumonisins (Gao et al., 2007; Battilani et al., 2018), but more susceptible to *A. flavus* and *A. nidulans* and aflatoxin production (Gao et al., 2009). Ogunola et al. (2017) observed that genes *ZmLOX1/2*, *5*, *8*, *9*, *10* and *12* fell under previously published QTL linked to a measurable reduction of aflatoxin in maize grains in one or more mapping populations. Moreover, association mapping results revealed 28 SNPs associated with reduced aflatoxin levels that fell within or near nine of the *ZmLOX* genes (Ogunola et al., 2017).

**Table 2:** Genes participating in the biosynthesis of oxylipins or their products and affecting mycotoxin contamination.

Genes/Compounds	Method	Functions	Crop/Fungus	References
*AnPPO*, *ZmLOX3* and *PnLOX2-3*	Fungal loss of function mutants	Production of sterigmatocystin	Maize/peanut/*Aspergillus nidulans*	Tsitsigiannis and Keller, 2006; Brodhagen et al., 2008
*ZmLOX3*	Plant loss of function mutants	Susceptibility to fumonisin contamination	Maize/*Fusarium verticillioides*	Gao et al., 2007; Battilani et al., 2018
*ZmLOX3*	Plant loss of function mutants	Resistance to aflatoxin contamination	Maize/*A. flavus*, *A. nidulans*	Gao et al., 2009
*AoLOXA*	Fungal loss of function mutants	Production of ochratoxin A	Wheat/*A. ochraceus*	Reverberi et al., 2010
*FvFUM1*, *FvLOX1*, *ZmLOX3*, *ZmLOX5* and *ZmLOX10*; fatty acids, oxylipins, and over 50 sphingolipids	Parent lines	Production of fumonisins	Maize/*F. verticillioides*	Lanubile et al., 2013; Beccaccioli et al., 2021
*ZmLOX12*	Plant loss of function mutants	Resistance to fumonisin contamination	Maize/*F. verticillioides*	Christensen et al., 2014
*LDS1*	Fungal loss of function mutants	Production of fumonisins	Maize/*F. verticillioides*	Scala et al., 2014
Oxylipins, sphingolipids, phospholipids, phytoceramides and amadori-glycated glycerophosphoethanolamines	Hybrids	Resistance to fumonisin contamination	Maize/*F. verticillioides*	Giorni et al., 2015; Righetti et al., 2021; Carbonell-Rozas et al., 2025
*AfPXG*	Fungal loss of function mutants	Production of aflatoxins	Maize/*A. flavus*	Hanano et al., 2015
*ZmLOX1/2*, *5*, *8*, *9*, *10* and *12*	Parent lines	Resistance to aflatoxin contamination	Maize/*A. flavus*	Ogunola et al., 2017
Lipoxygenase	Parent lines	Resistance to aflatoxin contamination	Peanut/*A. flavus*	Bhatnagar-Mathur et al., 2021
*ZmLOX4*	Plant loss of function and overexpressing mutants	Resistance to fumonisin contamination	Maize/*F. verticillioides*	Lanubile et al., 2021b; Guche et al., 2022; Ottaviani et al., 2026

FUM, fumonisin biosynthetic gene; LDS, linoleate diol synthase; LOX, lipoxygenase; PPO, (psi)-producing oxygenase; PXG: peroxygenase.

The antimicrobial activity of oxylipins was also experienced directly on fungi. For instance, the deletion of three *ppo* genes encoding fatty acid oxygenases in *A. nidulans* altered spore ratios and mycotoxin sterigmatocystin production, reducing the expression of genes involved in the biosynthesis of the latter one. Additionally, the Delta *ppo* mutants were defective in colonization of peanut seeds and the exogenous application of seed oxylipins to *Aspergillus* cultures hampered fungal sporulation and mycotoxin accumulation (Tsitsigiannis and Keller, 2006). To verify whether plant *LOX* genes could rescue *A. nidulans ppo* gene functionality, *ZmLOX3* was introduced into wild-type and Delta fungal strains. The expression of *ZmLOX3* favored conidia and sterigmatocystin produced amount in both backgrounds (Brodhagen et al., 2008). Moreover, when peanut seeds were infected by *A. nidulans* Delta *ppo* mutants the expression of *PnLOX2–3* was decreased suggesting a reciprocal oxylipin cross-talk in the *Aspergillus*-peanut pathosystem (Brodhagen et al., 2008). Similar findings were observed in *A. ochraceus*, where the *AoloxA* inhibition determined a different colony morphology, a delayed conidia formation, lower basal LOX activity, a limited content of 13-hydroperoxylinoleic acid and a considerable inhibition of ochratoxin A biosynthesis (Reverberi et al., 2010). Also, wheat seeds inoculated with the *AoloxA* mutant did not produce 9-hydroperoxylinoleic acid, known to be a crucial element in the host defense system, impairing the expression of the pathogenesis-related protein 1 (Reverberi et al., 2010). Comparable results were also found for *A. flavus*; indeed, silencing of the gene encoding a caleosin-like protein characterized by peroxygenase activity led to a reduced aflatoxin B_1_ production *in vitro*, a down-regulation of *aflR* and *aflD* genes and a compromised maize seed colonization (Hanano et al., 2015). On the other hand, when caleosin/peroxygenase-derived oxylipins were applied to *pxg* deficient strains they reestablished the wild-type phenotype (Hanano et al., 2015). More recently, proteomics analysis highlighted the production of lipoxygenase-mediated hydroperoxy fatty acids during *A. flavus*–peanut interaction (Bhatnagar-Mathur et al., 2021).

Contrasting observations were instead reported for *F. verticillioides*, where deletion of Linoleate Diol Synthase 1 (LDS1), one of the main enzymes responsible for oxylipin generation, caused a better growth, enhanced conidia and fumonisin production as well as an improved maize cob infection of *Fvlds1*-deleted mutants compared to its wild-type counterpart (Scala et al., 2014). In further study, when maize kernels were infected with a *F. verticillioides* fumonisin-deficient mutant and its wild-type strain, an alteration of plant lipidome was reported in presence of fumonisins along with a higher production of salicylic acid and JA (Beccaccioli et al., 2021). The impact of oxylipins on mycotoxin production is also mediated through changes in plant transcriptome. In this regard, Lanubile et al. (2013) found that the response of maize kernels to fumonisin-producing and nonproducing strains of *F. verticillioides* was different, and a delayed and weakened activation of defense and oxidative stress-related genes was displayed by the *fum1* mutant, presumably as a consequence of its reduced growth, compared to the wild-type strain. Unexpectedly, plant and fungal LOXs were up-regulated after *fum1* mutant inoculation (Lanubile et al., 2013), suggesting the presence of possible alternative strategies in the association between the polyketide synthase and the LOX activities. The presence of different polyketide synthase isoforms or the production of mycotoxins by additional pathways could be explored in this regard. Taken together, the evidence suggests that oxylipins participate in multiple layers of plant–fungus interactions. In summary, plant-derived oxylipins generally act as modulators of fungal growth and mycotoxin biosynthesis, often through cross-talk with JA-dependent signaling pathways.

## 5 CRISPR/Cas-based tools and oxylipins

Recently, clustered regularly interspaced short palindromic repeats (CRISPR) and its associated Cas protein has been widely applied to further explore the role of the enzymes involved in the oxylipin pathway (**Table 3**). In this regard, Pathi et al. (2020) used Cas endonuclease technology approach to generate loss of function mutations in *ZmLOX3*. After *Ustilago maydis* inoculation, *lox3* maize mutants showed reduced susceptibility and improved ROS accumulation implicating an enhanced defense response (Pathi et al., 2020). CRISPR/Cas9-induced knockout of *OsLOX1* in rice determined diminished tolerance to drought stress associated with elevated levels of H_2_O_2_ and malondialdehyde, and reduced expression and activities of the antioxidant enzymes compared with the wild-type (Weng et al., 2025). The targeted knockout of barley gene isoforms *LOXA* and *LOXC* enhanced grain storability proved by significantly higher germination rates, reduced lipid peroxidation, and improved seedling growth (Zeng et al., 2025). In cucumber (*Cucumis sativus* L.), density of glandular trichomes was severely impaired by the site-directed mutagenesis of a *CsLOX*, bringing also to a lower accumulation of JA and OPDA and down-regulation of genes related to glandular trichome development (Yang et al., 2025).

**Table 3:** Application of CRISPR/Cas-based tools for the editing of genes involved in the biosynthesis of oxylipins.

Genes	Functions	Crop	References
*OPR3* and *OPR7*	Male sterility, spikelet development	Arabidopsis, rice	You et al., 2019; Pak et al., 2021
*LOX3*	Resistance to *Ustilago maydis*	Maize	Pathi et al., 2020
*COI2a* and *COI2b*	Male sterility	Maize	Qi et al., 2022
*COI1a*, *COI1b* and *COI2*	Leaf senescence, male sterility, spikelet development, root growth, grain size and resistance against brown planthopper	Rice	Inagaki et al., 2023; Wang et al., 2023b
*LOX1*	Resistance to drought stress	Rice	Weng et al., 2025
*LOX*	Production of glandular trichomes	Cucumber	Yang et al., 2025
*LOXA* and *LOXC*	Improvement of grain storability	Barley	Zeng et al., 2025

COI, CORONATINE INSENSITIVE; LOX, lipoxygenase; OPR, oxo-phytodienoic acid reductase.

Besides *lipoxygenase* genes, also *OPR* were subjected to editing by CRISPR/Cas9 system. Male sterility was obtained by mutations of *OPR3* and *OPR7* genes in Arabidopsis and rice, respectively (Pak et al., 2021). Moreover, *Osopr7* knockout mutants showed reduced levels of endogenous JA and displayed an abnormal spikelet phenotype (You et al., 2019). Additionally, mutants for the COI-receptors that play a crucial role in the JA signaling pathway were generated in rice and maize, although with specific phenotypic effects (**Table 3**). It was observed that *OsCOI1b* gene regulates root growth and grain-size and performs similar activities with *OsCOI1a* in spikelet development, while *OsCOI2* controls leaf senescence, male sterility, root growth, and grain size (Inagaki et al., 2023; Wang et al., 2023b). Moreover, all *OsCOIs* contributed to the resistance against the brown planthopper *Nilaparvata lugens* (Inagaki et al., 2023; Wang et al., 2023b). Similar findings were reported for *ZmCOI2a* and *ZmCOI2b*, where *coi2a coi2b* maize double mutant showed non-dehiscent anthers, late anther development and male sterility (Qi et al., 2022).

Even though the use of these edited plant materials has been liberalized in some countries, regulatory issues still remain in others. Recently, a window of opportunity has opened up in Europe. Indeed, the Council of the European Union and the European Parliament have reached a political agreement that maintains a dedicated category for New Genomic Techniques (NGT)-1 plants considered equivalent to conventionally bred varieties. This legislation can mark the beginning of a new era for European agriculture, allowing researchers and breeders to more efficiently translate scientific advances into sustainable agricultural solutions.

## 6 Conclusion and future perspectives

As agriculture persists in dealing with climate change challenges, there are several points that require additional investigations. This includes exploring the connection between abiotic stressors (like drought and flooding), expected to increase with global warning, and susceptibility to fungal diseases and mycotoxin production. Therefore, there is an urgent need to foster climate-resilient plants with improved resistance to biotic stress. A wide array of plant oxylipins is generated in response to multiple physiological processes and environmental acclimatization. Their production starts from the oxygenation of polyunsaturated fatty acids and proceeds with the support of several downstream oxylipin biosynthetic enzymes as CYP74 enzymes. The papers collected in this review have presented some of the recent developments in oxylipin biology with particular emphasis on those involved in environmental stress and mycotoxin contamination. Despite in the last years the numerous efforts of authors to better understand oxylipin enzymes, some important questions remain to be explored. Several technical limitations in oxylipin detection were encountered. The low abundance, extreme structural diversity and inherent instability of these compounds often deriving from complex plant matrices as well as the lack of standardized protocols and internal standards require highly sensitive LC-MS/MS instruments. Future research using advanced technologies such as the CRISPR/Cas system and further omics approaches should overcome these challenges and shed light on oxylipin signaling and cross-talk. The most promising genotypes showing favorable traits could be easily included in breeding pipelines through crosses and targeted assisted selection in order to obtain pre-breeding material. The development of next generation sequencing technologies and high throughput phenomics platform will allow a more effective exploitation of large-scale breeding populations. Moreover, the possibility of employing these molecules as potential biological agents and resistance inductors by spraying them on crops could also be explored. Further investigation into the practical application of oxylipins as biostimulants or resistance inducers could significantly contribute to sustainable crop protection.
